# Perceived stress among nurses: A hospital-based study

**DOI:** 10.1192/j.eurpsy.2021.1061

**Published:** 2021-08-13

**Authors:** A. Hrairi, A. Kchaou, R. Masmoudi, A. Abbes, K. Jmal Hammami, M.L. Masmoudi, J. Masmoudi, M. Hajjeji

**Affiliations:** 1 The Department Of Occupational Medicine And Work-related Pathology, University hospital Hedi Chaker Sfax, SFAX, Tunisia; 2 Psychiatrie “a” Department, Hedi Chaker Hospital University -Sfax - Tunisia, sfax, Tunisia; 3 Department Of Occupational Medicine, HEDI CHAKER hospital, SFAX, Tunisia; 4 Occuaptional Department, HediChaker Hospital, sfax, Tunisia

## Abstract

**Introduction:**

Stress can be described as a dynamic and reciprocal relationship between the person and the environment. Nursing is considered as an occupation with a constellation of circumstances leading to stress.

**Objectives:**

This study aims to assess perceived stress among staff nurses in Hedi Chaker and Habib bourguiba Hospital from Sfax city, Tunisia

**Methods:**

Nurses from Hedi Chaker and Habib bourguiba University hospitals in Sfax- Tunisia were invited to complete a structured self-report questionnaire. The questionnaire consists of the following parts: Perceived Stress Scale (10-item form), personal data and information relevant to types of work shifts and years of experience.

**Results:**

A total of 146 (males = 49; females = 97) nurses participated in this study. The mean age was 37 years. Nearly 82 % of the participants considered themselves in very good health. Rotating shifts work was noted in 72.50% of cases. The average length of working experience was 7.96 years. The stress in most of nurses was in severe level (74.65%), followed by mild (23.28%) and moderate (2.05%) levels. High level of perceived stress was significantly associated with general health problems (P= 0.032). No significant association was found between level of perceived stress, types of work shifts and years of experience.

**Conclusions:**

The results show a significantly high level of stress among staff nurses. Hence, nurses need support and subsequent interventions to cope with stress. Actions in this direction may contribute to the improvement of health, well-being and quality of life of the professionals.

**Conflict of interest:**

No significant relationships.

